# Mosquito electrocuting traps for directly measuring biting rates and host-preferences of *Anopheles arabiensis* and *Anopheles funestus* outdoors

**DOI:** 10.1186/s12936-019-2726-x

**Published:** 2019-03-18

**Authors:** Felician C. Meza, Katharina S. Kreppel, Deodatus F. Maliti, Amos T. Mlwale, Nosrat Mirzai, Gerry F. Killeen, Heather M. Ferguson, Nicodem J. Govella

**Affiliations:** 1 Environmental Health and Ecological Sciences Department, Ifakara Health Institute, Off Mlabani Passage, P.O. Box 53, Ifakara, Tanzania; 2 Animal Health and Comparative Medicine, Institute of Biodiversity, University of Glasgow, Graham Kerr Building, Glasgow, G12 8QQ UK; 3 Bioelectronics Unit, University of Glasgow, Graham Kerr Building, Glasgow, G12 8QQ UK; 4 Vector Biology Department, Liverpool School of Tropical Medicine, Pembroke Place, Liverpool, L3 5QA UK

**Keywords:** Mosquito electrocuting trap, Human landing catch, Mosquitoes, Malaria, Host preference, *Anopheles arabiensis*, *Anopheles funestus*, Sampling, Human biting densities

## Abstract

**Background:**

Mosquito biting rates and host preferences are crucial determinants of human exposure to vector-borne diseases and the impact of vector control measures. The human landing catch (HLC) is a gold standard method for measuring human exposure to bites, but presents risks to participants by requiring some exposure to mosquito vectors. Mosquito electrocuting traps (METs) represent an exposure-free alternative to HLCs for measuring human exposure to malaria vectors. However, original MET prototypes were too small for measuring whole-body biting rates on humans or large animals like cattle. Here a much larger MET capable of encompassing humans or cattle was designed, and its performance was evaluated relative to both the original small MET and HLC and for quantifying malaria vector host preferences.

**Methods:**

Human landing catch, small human-baited METs (MET-SH), and large METs baited with either a human (MET-LH) or calves (MET-LC) were simultaneously used to capture wild malaria vectors outdoors in rural southern Tanzania. The four capture methods were compared in a Latin-square design over 20 nights. Malaria vector host preferences were estimated through comparison of the number of mosquitoes caught by large METs baited with either humans or cattle.

**Results:**

The MET-LH caught more than twice as many *Anopheles arabiensis* than either the MET-SH or HLC. It also caught higher number of *Anopheles funestus* sensu lato (s.l.) compared to the MET-SH or HLC. Similar numbers of *An. funestus* sensu stricto (s.s.) were caught in MET-LH and MET-SH collections. Catches of *An. arabiensis* with human or cattle-baited large METs were similar, indicating no clear preference for either host. In contrast, *An. funestus* s.s. exhibited a strong, but incomplete preference for humans.

**Conclusions:**

METs are a sensitive, practical tool for assessing mosquito biting rates and host preferences, and represent a safer alternative to the HLC. Additionally these findings suggest the HLC underestimate whole-body human exposure. MET collections indicated the *An. funestus* s.s. population in this setting had a higher than expected attack rate on cattle, potentially making eliminating of this species more difficult with human-targetted control measures. Supplementary vector control tools targetted at livestock may be required to effectively tackle this species.

## Background

Accurate estimation of mosquito biting rates and host preference are critical for assessing exposure risks of humans and animals to vector-borne diseases, and for optimizing the impact of vector control strategies [1–4]. Until relatively recently, measuring human exposure to mosquito bites outdoors necessitated laborious, potentially hazardous and ethically questionable human landing catches (HLCs) [5–7]. Despite these risks, the HLC remains the only technique considered reliable for direct estimation of human exposure to mosquito bites inside houses and outdoors, and over the course of the entire night [6, 8]. In addition to the limitations mentioned above, it is possible the HLC may also underestimate total human exposure to mosquito bites for a number of reasons. First, the HLC relies on the constant vigilance of the collectors throughout an entire night of sampling, and it is possible that capture efficiency drops as participants tire. Second, only mosquitoes attempting to feed on a person’s legs are collected with HLCs [6, 9]. While it is known that many malaria vectors are most attracted to the feet area [10–12], biting can occur on other parts of the body including the head and arms. While collectors may also attempt to capture mosquitoes landing on other parts of their body, these mosquitoes may go undetected given the focus on the lower leg area. Given the importance of measuring malaria transmission, there is a great need for surveillance tools that can accurately measure total human exposure to mosquito bites in both indoor and outdoor settings.

A new mosquito electrocuting trap (MET) has recently shown promise as a representative and safe alternative to the HLC for measuring human exposure to malaria vectors outdoors [13, 14]. This new tool produced similar estimates of relevant metrics of human exposure to malaria vectors (such as distribution of bites between indoors and outdoors and over the course of the night) as the HLC gold standard in urban Dar es Salaam [14]. Such measurements allow quantification of the proportion of human exposure occurring indoors and during hours when people are in bed, which are critical determinants of optimal deployment of bed nets, and other vector control tools [2, 4, 15]. Intervention choice should also be guided by the host preference of target mosquito vectors [2–4, 16]. For example, novel vector control approaches that target livestock with systemic insecticides [17, 18] will only be effective against vectors that commonly bite livestock as well as humans [2–4, 19].

Host preference is typically measured by comparing the number of mosquitoes attracted to different host types in a choice test [16]. However, such assays are often hard to implement under natural field settings due to the lack of standardized methods for sampling vectors attracted to humans and other animals. Consequently, the *human blood index* (HBI); defined as the proportion of blood meals that a vector species obtains from humans [20], is often used as a proxy for host choice instead of host preference. The HBI is estimated by sampling mosquitoes resting in and around houses [20–23], and identifying the source of their blood meal using molecular methods [24]. Although useful to confirm what mosquitoes actually feed on within a given environment, the HBI is dependent on the relative abundance of different host types and thus does not give an unbiased estimate of innate preference [16]. As it is difficult to measure preference across the range of potential hosts, HBI has been often used as a proxy of preference, even though it is only a measure of choice. Experimentally-controlled host preference assays, in which mosquitoes are given an equal opportunity to bite different hosts, provide the most reliable, direct and unambiguous measure of their innate behavioural preferences [25–28].

A range of field techniques have been proposed to estimate the host preference of mosquitoes [16, 29, 30]. These methods include a variety of net and stable-based traps that enclose either a human or animal host inside a physical structure, allowing mosquitoes to enter but impeding their exit [25]. Although useful, many of these methods have limitations that make them difficult to implement or interpret. For example, e-nets require a relatively large area for set up due to the requirement for a 10 m odour tube [30]. Other methods such as odour-baited entry traps (OBETs) involve luring mosquitoes to point source where they must enter a structure to be trapped. This requirement for entry behaviour may preferentially select endophilic (e.g. indoor biting) vector species; and thus not give a representative sample of outdoor biting mosquito taxa.

Electrocuting surfaces have been widely used to capture outdoor-biting tsetse flies [31], and have also been adapted for sampling host-seeking mosquitoes [13, 14, 30, 32–34]. A MET prototype previously applied to measure human exposure to malaria vectors across different times of the night performed consistently with the HLC gold standard method [14]. This original prototype was designed to operate similarly to HLC, with only mosquitoes approaching the lower leg area of a human bait being trapped [14]. Whilst this approach is appropriate when trying to replicate the estimates of human exposure gained from a HLC, it is not feasible for studies of mosquitoes host-seeking on other large animals as required to measure host preference. This study evaluated the performance of a new MET prototype designed to be capable of encompassing entire hosts (humans or livestock), and evaluated its performance relative to HLC and the original small sized MET for measuring human exposure to malaria vectors. Additionally large METs were used to measure the relative preference of African malaria vector species for biting human vs cattle hosts. In doing so, the versatility of MET-based sampling approaches to safely measure a range of epidemiologically-relevant mosquito vector ecological and behavioural traits were demonstrated.

## Methods

### Study area and site

The study was conducted in Sagamaganga village (S08°03.83′; E036°47.77′) [35–37], which is situated 15 km east of Ifakara town within the Kilombero Valley, south-eastern Tanzania. Most residents in this area live on subsistence farming, growing rice and maize as well as keeping livestock [38]. Despite the successful scale up of long lasting insecticide nets (LLINs), and undetectable level of the most historically-important vector *Anopheles gambiae* [39, 40], this area still experiences year-round malaria transmission [39, 40]. Most malaria transmission is mediated by *Anopheles funestus* sensu stricto (s.s.), a species that is thought to feed mainly inside house (endophilic) and on humans (anthrophilic) [41], and is highly physiologically resistant to pyrethroid insecticides [39]. *Anopheles arabiensis* is the most common and highly abundant anopheline species in the area but, but has a much lower rate of malaria infection than *An. funestus* s.s. [39].

### Trapping methods

#### Mosquito electrocuting trap (MET)

**Fig. 1:**
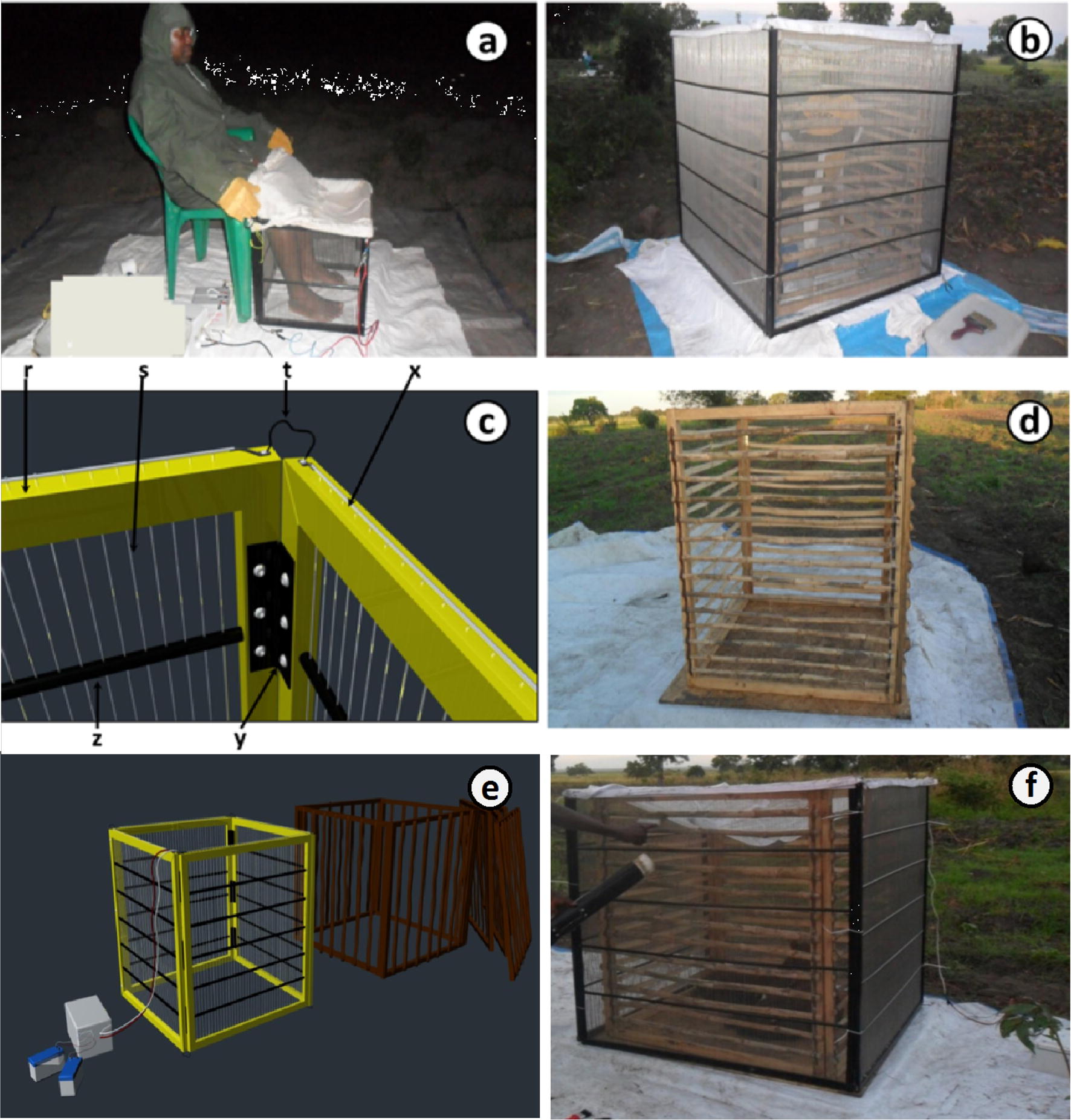
**a** Volunteer sampling mosquitoes by exposing his legs in the centre of the trap while the rest of his body is protected from mosquito bites. **b** Complete panels joined together to make a large Mosquito Electrocuting Trap (MET) to accommodates a sitting volunteer. **c** A 3-dimensional schematic of the MET showing panels made of PVC frame (r) with alternating stainless steel wire (s) arranged in parallel, spaced 5 mm apart, with drilled holes along the PVC frame (x), and supported with PVC struts (z) in the middle. The panels are interconnected by hinges (y), and electric wire joins positive and negative terminals of the panels (t). **d** Protective fence made of bamboo which is enclosed within the MET to prevent host contact with electrified grid. **e** A diagram shows the whole set up of a large MET. **f** Complete panels joined together to make a large MET-LC to accommodates cattle

The METs evaluated here operate in similar fashion to previous prototypes where electrocuting surfaces are placed around a host so as to intercept and kill mosquitoes as they attempt to land and bite [13, 14]. The MET prototypes used in early trials were made of 30 cm × 30 cm wooden panel frames, which could be assembled into a square box to encompass the lower legs of a seated human. The prototypes used here were made with PVC panel frames and held together with hinges, so that they were lighter and could be more easily assembled into a box shape (Fig. 1a). Two METsprototypes were used: one with the original frame size (30 cm^2^ panels) and a larger prototype with 125 cm × 122 cm panels, which could be assembled into a box to fully enclose the whole body of a sitting human volunteer or a standing/sleeping bovine calf (Fig. 1b). Both MET designs were composed of parallel stainless steel wires with alternating polarity, spaced 5 mm apart, and held in place by passing them through evenly-spaced holes drilled into the PVC frame (Fig. 1c). The large MET was fitted with a dry bamboo protective fence on the inside, so as to prevent any accidental contact of the volunteer or calf with the electrified wires (Fig. 1d). The top of the large MET was also covered with an untreated net, secured with Velcro^®^, to prevent any possible host exposure to mosquitoes flying in from above (Fig. 1b). During experiments, each MET (whether baited with a human or calves) was placed on a square wooden platform measuring 2 × 2 m, which was covered with a white cotton sheet to increase the visibility of the electrocuted mosquitoes to collectors. The MET is powered by 2 12 V batteries in parallel to make 24 V which produce a DC output that creates electric potential between alternating wires. The current–voltage combination has been optimized to kill mosquitoes on contact, but without damaging the specimen so it can be morphologically identified [42] as confirmed in previous studies [13, 14].

#### Human landing catch (HLC)

The HLC was performed by an adult male sitting on a chair and exposing his lower legs to mosquitoes. The volunteer continually inspected his legs using a torch for 45 min of each sampling hour. Any mosquitoes observed to land upon their legs during this time were collected using a mouth aspirator as previously described [43, 44].

### Study design

**Fig. 2:**
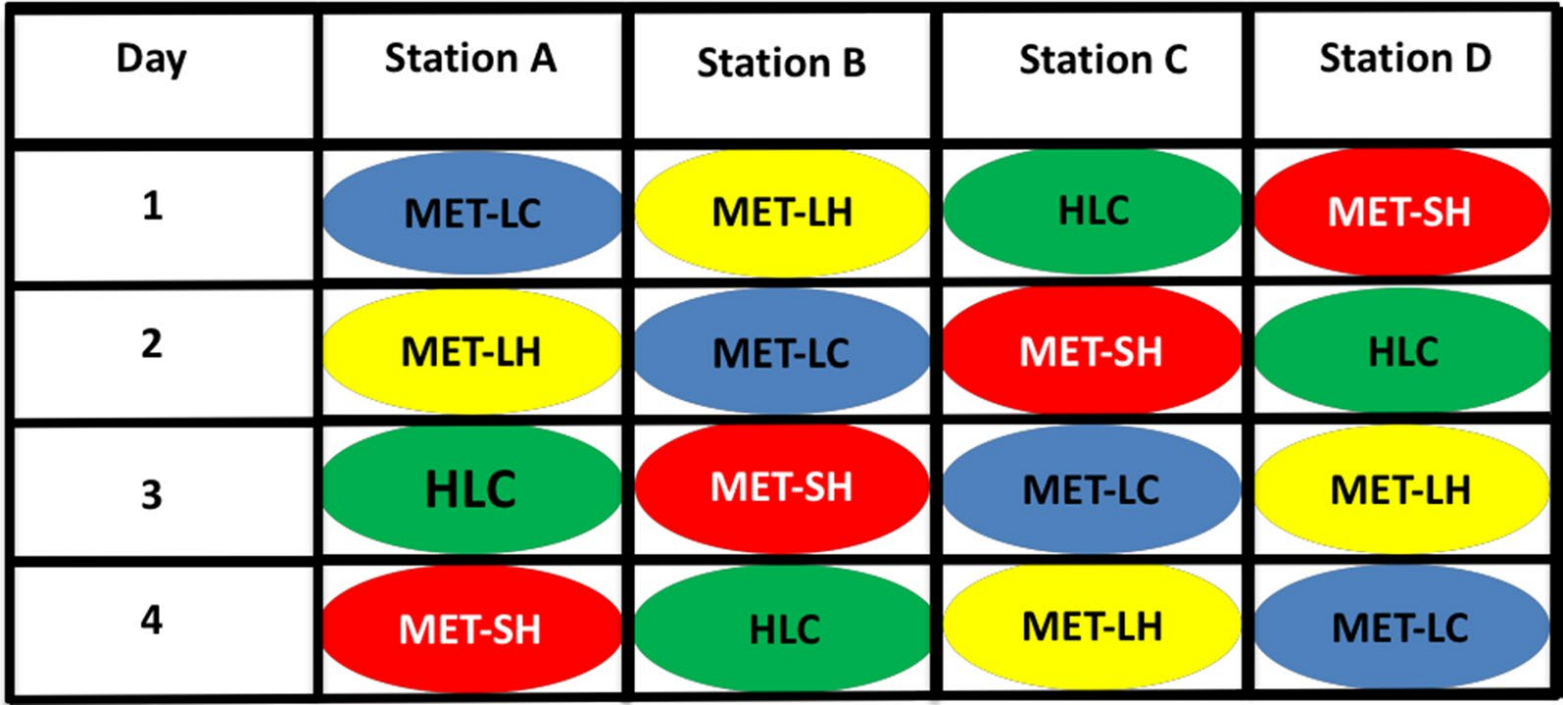
Schematic representation of a typical experimental design indicating 4 × 4 Latin Square with possible arrangements for one complete experimental rotation of capture methods. *MET-LC* large mosquito electrocuting trap baited with a cow (Blue), large mosquito electrocuting trap baited with a human (Yellow), *MET-SH* small mosquito electrocuting trap baited with a human (Red), *HLC* human landing catch (Green)

Experiments were conducted in an open field in Sagamaganga village. The field was bordered by two isolated compounds containing several houses, with one big cow shed on one side and a rice field on the other. Four sampling stations spaced approximately 20 m from each other along a straight line were set up in the field. Four different combinations of host and capture methods were evaluated: (1) a human volunteer conducting HLC, (2) a small MET with one adult male human (MET-SH), (3) a large MET containing one adult male human (MET-LH), and (4) a large MET baited with two female calves (~ 2 months old 60 to 80 kg average weight; MET-LC). Two calves were used as experience shows that calves are less stressed when kept together. The weight of male volunteers conducting HLC, MET-SH or MET-LH collections averaged ~ 65 kg. All four capture methods were used on each night of sampling, with one method allocated per sampling station. These capture techniques were then serially rotated through all four sampling stations in a 4 × 4 Latin square design (Fig. 2). Therefore, four nights were required to complete one replicate. The experiment was replicated five times, requiring a total of 20 sampling nights.

Each MET collection was made between 18:00 h and 6:00 h, with traps being run for 45 min of each hour. After the 45 min trapping period of each hour, MET traps were switched off and their outer surfaces and the white sheet below inspected for electrocuted mosquitoes. Mosquitoes found dead on the white sheet were collected using forceps, and those electrocuted and stuck to the surface panels were first swept using a small brush and collected by forceps. A pair of volunteers participated in each of the three human-baited sampling techniques. One volunteer would catch mosquitoes between 18:00–00:00 h, and the second between 00:00–06:00 h. Each pair of volunteers was randomly assigned to a particular station and remained associated with that station throughout the experiment, so that the systematic differences between individual station locations and volunteer pairs could be combined into a single source of variance. For the MET-LC, the same two female calves were systematically moved together with the large MET through all four sampling stations. Mosquitoes collected by different trapping methods were stored separately for each hour of collection in labelled paper cups. The voltage for the electrocuting trap was checked regularly.

### Mosquito processing

All mosquito specimens collected were sorted, counted and morphologically identified in the field with the aid of a dissection microscope [45]. Mosquitoes were identified as members of the *An. gambiae* complex (*An. gambiae* sensu lato), the *An. funestus* complex (*An. funestus* s.l.), *Anopheles coustani*, *Anopheles zeimanni*, *Culex* species, or *Aedes* species, and classified in terms of sex and abdominal status. A subsample of 1839 out of 15,322 of *An. gambiae* s.l. and all *An. funestus* s.l. (n = 2067) collected were stored individually in 1.5 ml eppendorf tubes containing desiccated silica gel covered with a small ball of cotton wool. Subsample of 7 *An. gambiae* s.l. from each trapping hour of each collection (MET and HLC) were used for individual species identification using molecular analysis. These 7 were haphazardly selected from the total collected each hour. In cases where less than 7 were collected in an hour, all were subsampled. This generated a subsample of 3680 *An. gambiae* s.l. of which approximately 50% were analysed by PCR for species identification (n = 1839 including representative samples from all trapping methods and dates [46, 47]. This subsample of individuals was analysed by enzyme-linked immune-absorbent assay (ELISA) for malaria sporozoite detection [48]. To avoid false positives, the ELISA lysates were heated in a boiling water bath for 10 min at 100 °C to inactivate heat-labile antigens other than *Plasmodium falciparum* circumsporozoite protein, which is not denaturable [49].

### Statistical analysis

Statistical analyses were carried out using the R statistical software version 3.0.2, augmented with the *matrix*, *lattice* and *lme4* packages [50]. Generalized linear mixed models (GLMMs) [51, 52] were used to estimate and compare the mean abundance of malaria vector species in nightly catches with different trap and host types. The number of mosquitoes caught per night was treated as the dependent variable, with trapping method fit as an independent fixed effect and sampling night and station fit as random effects. Models were fitted using a Poisson distribution. Likehood ratio tests were performed to test the significance of the main effect of trap type. Separate statistical models were fit for each malaria vector species.

To estimate the relative host preference of malaria vectors, only data from MET-LH and MET-LC were considered. Here comparisons were made between the proportion of malaria vectors caught in the human vs cattle baited traps. The response variable was defined as the relative proportion feeding on human-baited traps (MET-LH/[MET-LH + MET-LC]), with sampling night and station treated as random effects. These models were fitted with a binomial distribution with a logit link function. These host preference models did not include any fixed effect variables, with the estimated intercept representing host preference in terms of difference from the null hypothesis of an equal distribution of bites on human and calf baits. Separate analyses were performed for all the distinct species identified within the *An. funestus* s.l. Here count data were obtained by aggregating the total number of PCR-identified individuals from each species captured with each method in a single station on a single night. These aggregated count data were then analysed by GLMM as described above.

## Results

A total of 23,820 female *Anopheles* mosquitoes were captured, of which most were *An. gambiae* s.l. (Table 1). The next most abundant mosquito genus was *Culex*. (Table 1). A subsample of 1839 specimens from the *An. gambiae* complex were tested by PCR, out of which 1644 (89%) were successfully amplified. All of these *An. gambiae* s.l. were confirmed to be *An. arabiensis*. Therefore, from this point onward, all results obtained for *An. gambiae* s.l. are considered representative of *An. arabiensis* and referred to as such.

**Table 1:** The total number and type of mosquitoes captured by the human landing catch (HLC), small size mosquito electrocuting trap (MET-SH), large size mosquito electrocuting trap baited with human (MET-LH, large size mosquito electrocuting trap baited with cow (MET-LC)

Species	Capture method					
	HLC	MET-SH	MET-LH	MET-LC	Total catch	%
*Anopheles* spp.						
*Anopheles arabiensis*^a^	1824	2331	5631	5536	15,322	39.8
*Anopheles funestus* s.s.	116	227	304	109	756	2.0
*Anopheles rivulorum*	55	38	49	136	278	0.7
*Anopheles leesoni*	11	4	27	22	64	0.2
*Anopheles funestus* s.l.^b^	115	277	359	217	968	2.5
*Anopheles coustani*	923	328	713	1330	3294	8.6
*Anopheles ziemanni*	258	220	472	896	1846	4.8
*Anopheles pharoensis*	118	86	182	193	579	1.5
*Anopheles squamosus*	63	61	145	805	1075	2.8
*Anopheles maculpalpis*	5	0	1	0	6	0.01
*Anopheles wellcomei*	87	66	153	300	606	1.6
*Other mosquito* spp.						
*Culex* spp.	1573	1012	4078	5673	12,336	32.0
*Mansonia* spp.	375	242	433	267	1317	3.4
*Coquillettidia*	7	21	32	12	72	0.2
*Aedes* spp.	4	0	0	1	5	0.01

^a^Originally identified morphologically as *An. gambiae* s.l. and then confirmed to be 100% *An. arabiensis* by PCR (All 1644 successfully amplified specimens)

^b^*An. funestus* s.l. which could not be identified to species because did not amplify

PCR amplification was successful for only about half of *An. funestus* s.l. specimens (1098/2066). Within these samples, *An. funestus* s.s. was the most prevalent 69% (756/1098), followed by *Anopheles rivulorum* 25% (278/1098) and *Anopheles leesoni* 6% (46/1098) (Table 1). The proportion of *An. funestus* s.l. whose DNA could be successfully amplified varied between trapping methods as follows: HLC = 61% (n_total_ = 297), MET-SH = 49% (n_total_ = 546), MET-LH = 51% (n_total_ = 39) and MET-LC = 56% (n_total_ = 484). Amplification rates from specimens collected in HLC were significantly higher than from MET-SH (χ_1_^2^ = 11.16, P < 0.001) and the MET-LH (χ_1_^2^ = 8.30, df = 1, P < 0.001). Amplification rates were similar in *An. funestus* s.l. collected in HLC and MET-LC (χ_1_^2^ = 2.81, P = 0.09). There was no significant differences in *An. funestus* s.l. amplification rates between any of the 3 MET types (P > 0.05 in all cases).

The proportion of *An. arabiensis* and PCR- confirmed *An. funestus* s.s. infected with sporozoites were 0.18% (3/1644) and 0.27% (2/756) respectively. None of the other sibling species from the *An. funestus* group or unidentified *An. funestus* s.l. were found to be infected with sporozoites.

**Fig. 3:**
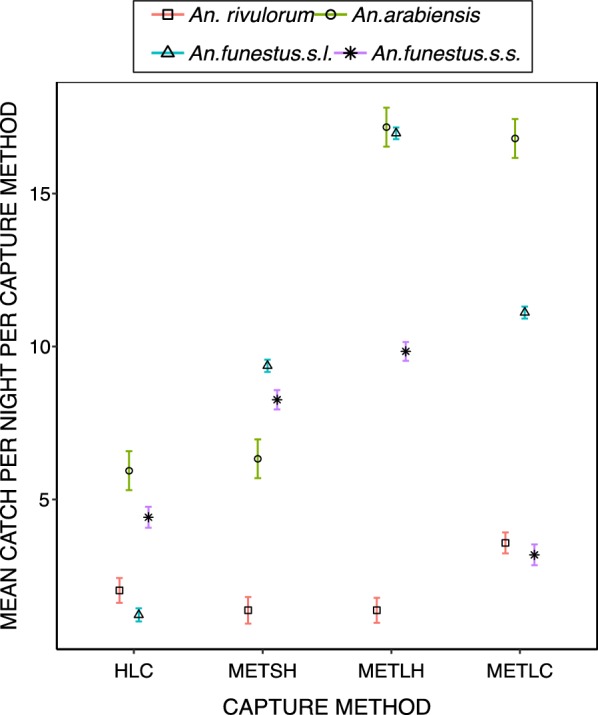
Estimated attack rates of individual *Anopheles* species per night per capture method (mean and 95% confidence intervals, as estimated from fitting a Poisson-distributed generalized linear mixed model with no intercept)

The MET-SH sampled a similar number of *An. arabiensis* as the HLC (Table 2). The MET-LH caught more than twice as many *An. arabiensis* per night as either HLC (RR = 2.89, *P *<0.001) or MET-SH (Tables 1, 2, Fig. 3). The MET-SH captured two times more *An. funestus* s.l. than HLC (Table 2), while the MET-LH consistently caught more *An. funestus* s.l. as either the HLC (RR = 3.63, *P *< 0.001) or MET-SH (Tables 1, 2, Fig. 3). However, MET-LH caught similar numbers of *An. funestus* s.s. and *An. rivulorum* as MET-SH (Table 2, Fig. 3).

**Table 2:** Comparisons of the mean number of females of mosquito species caught per night by HLC, MET-LH, MET-LC relative to reference mosquito electrocuting trap (MET-SH)

Capture Method	Mean catch	RR [95% CI]	P value
*Anopheles arabiensis*			
HLC	5.94	0.93 [0.88, 0.99]	0.049
MET-SH	6.33	1^a^	NA
MET-LH	17.17	2.71 [2.57, 2.85]	< 0.001
MET-LC	16.80	2.65 [2.52, 2.79]	< 0.001
*Anopheles funestus* s.s.			
HLC	4.42	0.53 [0.42, 0.68]	< 0.001
MET-SH	8.26	1^a^	NA
MET-LH	9.84	1.19 [0.99, 1.42]	0.053
MET-LC	3.19	0.38 [0.31, 0.48]	< 0.001
*Anopheles rivulorum*			
HLC	2.03	1.48 [0.97, 2.25]	0.069
MET-SH	1.38	1^a^	NA
MET-LH	1.38	1.00 [0.65, 1.53]	0.996
MET-LC	3.58	2.61 [1.81, 3.75]	< 0.001
*Anopheles funestus* s.l.			
HLC	1.23	0.49 [0.43, 0.57]	< 0.001
MET-SH	9.37	1^a^	NA
MET-LH	16.97	1.81 [1.64, 2.01]	< 0.001
MET-LC	11.11	1.19 [1.06, 1.32]	0.003

Results are based on 20 nights of collection with each trap type

*RR* relative rate, *CI* confidence interval, *NA* not applicable because it is a reference capture method

^a^Reference value

**Fig. 4:**
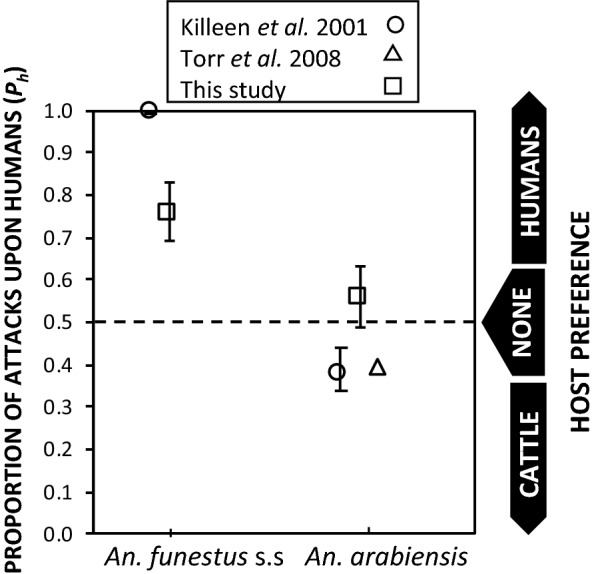
Estimated proportion of attacks on humans versus cattle when offered a choice between one of each host species (mean and 95% confidence intervals) for *Anopheles funestus* s.s. and *Anopheles arabiensis* in Segera, northern Tanzania [22], and Zimbabwe (data extracted from Fig. 7 in reference [30]) compared to those obtained by this study in southern Tanzania. The estimated proportion of attacks on humans (*P*_*h*_) was calculated as follows for the historical Tanzanian example, based on published estimates for the relative availability of humans versus cattle (*λ*): *P*_*h*_ = 1/(1 + *λ*) [61]

On the basis of the numbers of mosquitoes caught in large METs baited with different host types, *An. arabiensis* was estimated to be attracted to human and cattle hosts at a similar rate (Table 3, Fig. 4), indicating this vector population has no distinct preference for either host type. In contrast, *An. funestus* s.s. exhibited a strong preference for humans (76%, see Table 3, Fig. 4), whereas *An. rivulorum* preferred cattle (83%, Table 3, Fig. 4).

**Table 3:** Proportion of attack of *Anopheles* species on human showing the 95% confidence interval around the preference estimates as were observed from host seeking MET-LC and MET-LH as estimated by binary logistic GLMM regression

*Anopheles* species	*P*_*h*_	95% CI	Z value	P value
*An. arabiensis*	0.56 (11,167)	[0.43–0.67]	0.937	0.349
*An. funestus* s.s.	0.76 (413)	[0.68–0.82]	5.85	0.001
*An. rivulorum*	0.27 (185)	[0.18–0.37]	0.48	0.001

In the *P*_*h*_ column, numbers in bracket represent the denominator (e.g. total number of caught host seeking on humans and cattle combined)

*P*_*h*_ is the proportion of attack on human

## Discussion

Two sizes of electrocuting traps were evaluated for assessment of the biting densities and host preference of afrotropical malaria vectors in rural Tanzania. The large prototype baited with humans captured greater numbers of both *An. arabiensis* and *An. funestus* s.l. than the smaller prototype and the existing HLC gold standard. The large METs also proved effective for sampling mosquitoes attracted to cattle and human hosts. Estimates of host preference from MET collections indicated *An. arabiensis* is attracted to human and cattle hosts at a similar rate with no clear preference, whereas *An. funestus* s.s. clearly preferred humans over cattle. In contrast, *An. rivulorum* strongly preferred cattle. Notably, estimates of human biting rates in *An. arabiensis* and *An. funestus* s.l. were considerably greater when derived from the large MET than the small MET or the HLC. This raises the possibility that total human exposure to malaria vectors may be underestimated by current HLC gold standard method. The enhanced performance of large MET, is presumably due to the fact that, it samples a greater surface area around the host by encompassing their entire body and not just their lower legs. Such differences in sampling performance between small vs large METs were not detected for less abundant malaria vector species (*An. funestus* s.s. and *An. rivulorum)* [39]. However, this may be due to reduced statistical power to detect differences in these groups rather than a difference in their response to traps of different size.

Although the HLC is considered a gold standard approach for measuring human biting rates by mosquitoes, these results suggest it may underestimate total human exposure to *Anopheles* vector bites [43, 53, 54] and thus malaria transmission [55–57]. However, this hypothesis must be further investigated because it remains unclear whether all mosquitoes trapped with the large MET were actively host seeking, or whether some proportion was trapped during random or otherwise non-host directed flying. Ideally this could have been evaluated here by comparing the number of *An. gambiae* s.l. collected in baited and unbaited MET collections. Although this was not possible in this study due to limitations in the numbers of METs available and time period for study, its encouraged to be investigated in the future. In summary, the results indicate that METs are efficacious for estimating both malaria vector biting rates and their host preference in outdoor environments.

This study also demonstrates that the MET can be practically applied to quantify vector host preferences by increasing its size so it can accomodate large non-human hosts. Many existing methods for assessing host preference rely on luring mosquitoes towards the odour component of host stimuli [28, 32, 58, 59]. Although useful, these approaches may fail to capture the full range of visual, odour, heat and other stimuli arising from a host individual. The large MET evaluated here overcomes these limitations by presenting hosts to mosquitoes in a relatively natural way, with mosquitoes attempting to feed being intercepted just before they land. By increasing the size of the MET so it can encompass an entire host, this tool has potential to be applied to assess mosquito biting rates on a range of hosts including wildlife and domestic animals. This could be particularly useful to study the transmission of mosquito-borne zoonotic diseases [60]. The MET prototypes assessed here were also found to be reasonably practical for field use. These METs are made of durable but lightweight materials that are stackable, compact and easy to assemble.

Despite their advantages, METs also have practical limitations. The most notable limitation is the MET’s reliance on electrical components which may break or short circuit if exposed to excessive moisture. However, this specific limitation can be overcome by placement of traps under a tarpaulin cover and on platform which would enable their use even during the rainy seasons as exemplified in Fig. 1b of the study by Maliti and colleagues [13]. Furthermore, the METs used here are currently research prototypes that are produced individually by the Bioelectronics Department at the University of Glasgow, UK. The Ifakara Health Institute and the University of Glasgow have submitted a joint UK patent application for the MET (application number 1708369.2) which is currently under review. This technology is available for licencing through an Easy Access IP agreement, with design details available on request from the University of Glasgow. The costs of bespoke production per unit on this basis are currently quite high, ~ £1100 for a large MET, which is too high for most routine vector density surveillance applications. However the costs of these devices are anticipated to be significantly lower if produced in volume and could become much more economically viable in the future.

Use of METs has helped to confirm key aspects of the feeding behaviour of the main malaria vectors in the Kilombero Valley, and shed light on their ecology and potential response to control. For example, *An funestus* s.s. was confirmed as having a preference for humans over cattle, in contrast to *An. arabiensis* which was attracted to humans and cattle at a similar rate. A previous study in Zimbabwe using e-nets baited with host odour [30] and a modelling analysis of host demography and choice data from northern Tanzania [61] indicate that *An. arabiensis* had a modest preference for cattle over humans. The lack of preference for cattle in this study may be due to the use of relatively small calves, rather than full-grown adults [30] or entire herds of all ages [61]. Variation in biomass and attractiveness to mosquitoes with age and pregnancy have such strong influence upon human exposure that these demographic factors play a defining role in shaping malaria burden distribution across at-risk populations [62–65]. Evaluation of potential changes in mosquito host preference over time or between sites should ideally use consistent trapping methods with standardized host density and biomass or standardized synthetic odour lures.

Use of METs also helped update and further elucidate the ecology of *An. funestus* s.l. vectors in Kilombero. Historically, this species group has received relatively little attention in southern Tanzania, but may now be the most important vector of persisting transmission [39]. The species composition of the *An. funestus* s.l. reported here is consistent with other studies in the Kilombero valley [39, 40], with *An. funestus* s.s. being the most prevalent, followed by *An. rivulorum* and *An. leesoni*. However, only about half of *An. funestus* s.l. could be successfully identified to species level. The much lower amplification rates for *An. funestus* s.l. than for *An. gambiae* s.l. is most probably due to restricted availability of primers (only available for 4 of the 9 species in the r group). The lack of amplification of some *An. funestus* s.l. could indicate the presence of other species within the *An. funestus* group. On the basis of samples that could be identified, these results indicate *An. funestus* s.s. has a strong preference for humans over cattle as consistent with previous studies [22, 41, 66]. However, the degree of anthropophily in *An. funestus* s.s. found here is somewhat lower compared than previously reported in northern Tanzania where this species was estimated to feed almost entirely on humans [22]. The results of this present study are consistent with more recent reports from Zambia where the human attack rate of *An. funestus* was only 41.2% [27], and another in western Kenya where the human blood index of *An. funestus* was only 60% [21]. These results indicate that *An. funestus* s.s. in the Kilombero Valley and elsewhere in Africa can exploit cattle as a source of blood. Therefore, this species may be more difficult to eliminate with LLINs and indoor residual spray (IRS). Thus, complementary interventions that target livestock as alternative blood sources may also be required to tackle this species.

## Conclusions

This study adds to a growing body of evidence that, METs are a sensitive, practical, exposure-free alternative to the HLC gold standard tool for assessing human biting rates and measuring host preferences. Estimates of malaria vector biting rates were considerably higher in large METs, suggesting that total human exposure to bites may be underestimated by conventional methods. METs showed a higher than expected preference upon cattle for *An. funestus* s.s. suggesting that supplementary interventions may be needed to tackle this important vector.

## Abbreviations

HLC: human landing catch
MET-SH: small mosquito electrocuting trap baited with human volunteer
MET-LH: large mosquito electrocuting trap baited with human volunteer
MET-LC: large cattle-baited mosquito electrocuting trap
HBI: human blood index
LLINs: long-lasting insecticide nets
IRS: indoor residual spray
PCR: polymerase chain reaction
ELISA: enzyme-linked immune-absorbent assay
GLMMs: generalized linear mixed models

## References

[1] Lyimo IN, Ferguson HM (2009). Ecological and evolutionary determinants of host species choice in mosquito vectors. Trends Parasitol..

[2] Killeen GF (2014). Characterizing, controlling and eliminating residual malaria transmission. Malar J..

[3] Killeen GF, Kiware SS, Okumu FO, Sinka ME, Moyes CL, Massey NC (2017). Going beyond personal protection against mosquito bites to eliminate malaria transmission: population suppression of malaria vectors that exploit both human and animal blood. BMJ Glob Health..

[4] Killeen GF, Seyoum A, Gimnig JE, Stevenson JC, Drakeley CJ, Chitnis N (2014). Made-to-measure malaria vector control strategies: rational design based on insecticide properties and coverage of blood resources for mosquitoes. Malar J..

[5] Killeen G, Ross A, Smith T (2006). Infectiousness of malaria-endemic human populations to vectors. Am J Trop Med Hyg.

[6] Achee NL, Gould F, Perkins TA, Reiner RC, Morrison AC, Ritchie SA (2015). A critical assessment of vector control for dengue prevention. PLoS Negl Trop Dis..

[7] Seyoum A, Sikaala CH, Chanda J, Chinula D, Ntamatungiro AJ, Hawela M (2012). Human exposure to anopheline mosquitoes occurs primarily indoors, even for users of insecticide- treated nets in Luangwa Valley, South-east Zambia. Parasit Vectors..

[8] Lima JBP, Rosa-Freitas MG, Rodovalho CM, Santos F, Lourenço-de-Oliveira R (2014). Is there an efficient trap or collection method for sampling *Anopheles darlingi* and other malaria vectors that can describe the essential parameters affecting transmission dynamics as effectively as human landing catches?-A review. Mem Inst Oswaldo Cruz.

[9] Mboera LEG (2005). Sampling techniques for adult Afrotropical malaria vectors and their reliability in the estimation of entomological inoculation rate. Tanzan J Health Res..

[10] De Jong R, Knols BGJ (1995). Selection of biting sites on man by two malaria mosquito species. Experientia.

[11] Dekker T, Takken W, Knols BGJ, Bouman E, van de Laak S, de Bever A (1998). Selection of biting sites on a human host by *Anopheles gambiae* s.s., *An. arabiensis* and *An. quadriannulatus*. Entomol Exp Appl.

[12] Takken W, Knols BGJ (1999). Odor-mediated behavior of Afrotropical malaria mosquitoes. Annu Rev Entomol.

[13] Maliti DV, Govella NJ, Killeen GF, Mirzai N, Johnson PCD, Kreppel K (2015). Development and evaluation of mosquito-electrocuting traps as alternatives to the human landing catch technique for sampling host-seeking malaria vectors. Malar J..

[14] Govella NJ, Maliti DF, Mlwale AT, Masallu JP, Mirzai N, Johnson PCD (2016). An improved mosquito electrocuting trap that safely reproduces epidemiologically relevant metrics of mosquito human-feeding behaviours as determined by human landing catch. Malar J..

[15] Huho B, Briët O, Seyoum A, Sikaala C, Bayoh N, Gimnig J (2013). Consistently high estimates for the proportion of human exposure to malaria vector populations occurring indoors in rural Africa. Int J Epidemiol.

[16] Takken W, Verhulst NO (2013). Host preferences of blood-feeding mosquitoes. Annu Rev Entomol.

[17] Lyimo IN, Kessy ST, Mbina KF, Daraja AA, Mnyone LL (2017). Ivermectin-treated cattle reduces blood digestion, egg production and survival of a free-living population of *Anopheles arabiensis* under semi-field condition in south-eastern Tanzania. Malar J..

[18] Pooda HS, Rayaisse JB, Hien DFDS, Lefèvre T, Yerbanga SR, Bengaly Z (2015). Administration of ivermectin to peridomestic cattle: a promising approach to target the residual transmission of human malaria. Malar J..

[19] Kiware SS, Chitnis N, Devine GJ, Moore SJ, Majambere S, Killeen GF (2012). Biologically meaningful coverage indicators for eliminating malaria transmission. Biol Lett.

[20] Garrett-Jones C (1964). The human blood index of malaria vectors in relation to epidemiological assessment. Bull World Health Organ.

[21] Degefa T, Yewhalaw D, Zhou G, Lee MC, Atieli H, Githeko AK (2017). Indoor and outdoor malaria vector surveillance in western Kenya: implications for better understanding of residual transmission. Malar J..

[22] White GB, Magayuka SA (1972). Boreham PFL. Comparative studies on sibling species of the *Anopheles gambiae* Giles complex (Dipt., Culicidae): bionomics and vectorial activity of species A and species B at Segera, Tanzania. Bull Entomol Res.

[23] Kulkarni MA, Kweka E, Nyale E, Lyatuu E, Chandramohan D, Rau ME (2006). Entomological evaluation of malaria vectors at different altitudes in Hai District, Northeastern Tanzania. J Med Entomol..

[24] Beier JC, Perkins PV, Wirtz RA, Koros J, Diggs D, Gargan TP (1988). Bloodmeal identification by direct enzyme-linked immunosorbent assay (ELISA), tested on Anopheles (Diptera: Culicidae) in Kenya. J Med Entomol.

[25] Mery F, Burns JG (2010). Behavioural plasticity: an interaction between evolution and experience. Evol Ecol.

[26] Lobo NF, St. Laurent B, Sikaala CH, Hamainza B, Chanda J, Chinula D (2015). Unexpected diversity of Anopheles species in Eastern Zambia: implications for evaluating vector behavior and interventions using molecular tools. Sci Rep..

[27] Laurent B, Burton TA, Zubaidah S, Miller HC, Asih PB, Baharuddin A (2017). Host attraction and biting behaviour of Anopheles mosquitoes in South Halmahera, Indonesia. Malar J..

[28] Lefèvre T, Gouagna LC, Dabiré KR, Elguero E, Fontenille D, Renaud F (2009). Beyond nature and nurture: phenotypic plasticity in blood-feeding behavior of *Anopheles gambiae* s.s. when humans are not readily accessible. Am J Trop Med Hyg.

[29] Silver JB, Service MW (2008). Mosquito ecology: field sampling methods.

[30] Torr SJ, Della Torre A, Calzetta M, Costantini C, Vale GA (2008). Towards a fuller understanding of mosquito behaviour: use of electrocuting grids to compare the odour-orientated responses of Anopheles arabiensis and An. quadriannulatus in the field. Med Vet Entomol.

[31] Vale GA (1974). Attractants for controlling and surveying tsetse populations. Trans R Soc Trop Med Hyg.

[32] Hawkes F, Manin BO, Ng SH, Torr SJ, Drakeley C, Chua TH (2017). Evaluation of electric nets as means to sample mosquito vectors host-seeking on humans and primates. Parasit Vectors..

[33] Knols BGJ, Mboera LEG, Takken W (1998). Electric nets for studying odour-mediated host-seeking behaviour of mosquitoes. Med Vet Entomol.

[34] Majambere S, Masue D, Mlacha Y, Govella NJ, Magesa SM, Killeen GF (2013). Advantages and limitations of commercially available electrocuting grids for studying mosquito behaviour. Parasit Vectors..

[35] Kreppel KS, Johnson PCD, Govella NJ, Pombi M, Maliti D, Ferguson HM (2015). Comparative evaluation of the Sticky-Resting-Box-Trap, the standardised resting-bucket-trap and indoor aspiration for sampling malaria vectors. Parasit Vectors..

[36] Main BJ, Lee Y, Ferguson HM, Kreppel KS, Kihonda A, Govella NJ (2016). The genetic basis of host preference and resting behavior in the major African malaria vector, *Anopheles arabiensis*. PLoS Genet..

[37] Mayagaya VS, Nkwengulila G, Lyimo IN, Kihonda J, Mtambala H, Ngonyani H (2015). The impact of livestock on the abundance, resting behaviour and sporozoite rate of malaria vectors in southern Tanzania. Malar J..

[38] Schellenberg JRA, Abdulla S, Nathan R, Mukasa O, Marchant TJ, Kikumbih N (2001). Effect of large-scale social marketing of insecticide-treated nets on child survival in rural Tanzania. Lancet.

[39] Kaindoa EW, Matowo NS, Ngowo HS, Mkandawile G, Mmbando A, Finda M (2017). Interventions that effectively target *Anopheles funestus* mosquitoes could significantly improve control of persistent malaria transmission in south-eastern Tanzania. PLoS ONE.

[40] Lwetoijera DW, Harris C, Kiware SS, Dongus S, Devine GJ, McCall PJ (2014). Increasing role of *Anopheles funestus* and *Anopheles arabiensis* in malaria transmission in the Kilombero Valley, Tanzania. Malar J..

[41] Gillies MT, De Meillon B (1968). The Anophelinae of Africa south of the Sahara (Ethiopian zoogeographical region).

[42] Maliti D V. Ecological and genetic determinants of malaria vectors feeding and resting behaviours. Ph.D. Thesis. University of Glasgow; 2015.

[43] Govella NJ, Chaki PP, Geissbuhler Y, Kannady K, Okumu F, Charlwood JD (2009). A new tent trap for sampling exophagic and endophagic members of the *Anopheles gambiae* complex. Malar J..

[44] Gimnig JE, Walker ED, Otieno P, Kosgei J, Olang G, Ombok M (2013). Incidence of malaria among mosquito collectors conducting human landing catches in western Kenya. Am J Trop Med Hyg.

[45] Gillies MT, Coetzee M (1987). A supplement to the Anophelinae of Africa South of the Sahara. Publ S Afr Inst Med Res..

[46] Scott JA, Brogdon WG, Collins FH (1993). Identification of single specimens of the *Anopheles gambiae* complex by the polymerase chain reaction. Am J Trop Med Hyg.

[47] Koekemoer LL, Kamau L, Hunt RH, Coetzee M (2002). A cocktail polymerase chain reaction assay to identify members of the *Anopheles funestus* (Diptera: Culicidae) group. Am J Trop Med Hyg.

[48] Beier JC, Perkins PV, Koros JK, Onyango FK, Gargan TP, Wirtz RA (1990). Malaria sporozoite detection by dissection and ELISA to assess infectivity of afrotropical Anopheles (Diptera: Culicidae). J Med Entomol.

[49] Durnez L, Van Bortel W, Denis L, Roelants P, Veracx A, Trung HD (2011). False positive circumsporozoite protein ELISA: a challenge for the estimation of the entomological inoculation rate of malaria and for vector incrimination. Malar J..

[50] Bates D, Mächler M, Bolker BM, Walker SC (2015). Fitting linear mixed-effects models using lme4. J Stat Softw..

[51] Crawley MJ (2007). The R Book.

[52] Bolker BM, Brooks ME, Clark CJ, Geange SW, Poulsen JR, Stevens MHH (2009). Generalized linear mixed models: a practical guide for ecology and evolution. Trends Ecol Evol.

[53] Russell TL, Govella NJ, Azizi S, Drakeley CJ, Kachur SP, Killeen GF (2011). Increased proportions of outdoor feeding among residual malaria vector populations following increased use of insecticide-treated nets in rural Tanzania. Malar J..

[54] Briët OJT, Huho BJ, Gimnig JE, Bayoh N, Seyoum A, Sikaala CH (2015). Applications and limitations of Centers for Disease Control and Prevention miniature light traps for measuring biting densities of African malaria vector populations: a pooled-analysis of 13 comparisons with human landing catches. Malar J..

[55] Beier JC, Killeen GF, Githure JI (1999). Entomologic inoculation rates and *Plasmodium falciparum* malaria prevalence in Africa. Am J Trop Med Hyg.

[56] Kelly-Hope LA, McKenzie FE (2009). The multiplicity of malaria transmission: a review of entomological inoculation rate measurements and methods across sub-Saharan Africa. Malar J..

[57] Shaukat AM, Breman JG, McKenzie FE (2010). Using the entomological inoculation rate to assess the impact of vector control on malaria parasite transmission and elimination. Malar J..

[58] Mahande A, Mosha F, Mahande J, Kweka E (2007). Feeding and resting behaviour of malaria vector, *Anopheles arabiensis* with reference to zooprophylaxis. Malar J..

[59] Costantini C, Sagnon N, Della Torre A, Diallo M, Brady J, Gibson G (1998). Odor-mediated host preferences of West African mosquitoes, with particular reference to malaria vectors. Am J Trop Med Hyg.

[60] Wong ML, Chua TH, Leong CS, Khaw LT, Fornace K, Wan-Sulaiman W-Y (2015). Seasonal and spatial dynamics of the primary vector of *Plasmodium knowlesi* within a major transmission focus in Sabah, Malaysia. PLoS Negl Trop Dis..

[61] Killeen GF, McKenzie FE, Foy BD, Bøgh C, Beier JC (2001). The availability of potential hosts as a determinant of feeding behaviours and malaria transmission by African mosquito populations. Trans R Soc Trop Med Hyg.

[62] Port GR, Boreham PFL, Bryan JH (1980). The relationship of host size to feeding by mosquitoes of the *Anopheles gambiae* Giles complex (Diptera: Culicidae). Bull Entomol Res.

[63] Smith T, Maire N, Dietz K, Killeen GF, Vounatsou P, Molineaux L (2006). Relationship between the entomologic inoculation rate and the force of infection for *Plasmodium falciparum* malaria. Am J Trop Med Hyg.

[64] Smith T, Killeen G, Lengeler C, Tanner M (2004). Relationships between the outcome of *Plasmodium falciparum* infection and the intensity of transmission in Africa. Am J Trop Med Hyg.

[65] Lindsay S, Ansell J, Selman C, Cox V, Hamilton K, Walraven G (2000). Effect of pregnancy on exposure to malaria mosquitoes. Lancet.

[66] Kiszewski A, Mellinger A, Spielman A, Malaney P, Sachs SE, Sachs J (2004). A global index representing the stability of malaria transmission. Am J Trop Med Hyg.

